# 

**Published:** 1880-02-20

**Authors:** 


### EDITORIAL AND MISCELLANEOUS.

*Cinchonia Alkaloid.*—We invite attention to advertisement of Cinchonia Alkaloid, by Messrs. Powers & Weightman.

*Petrolina and Petrolina Oil.*—These are valuable articles. The Petrolina never gets rancid and is excellent as a pomade for the hair, as a bland, mild application to sores and ulcers, and a splendid vehicle for ointments, salves, etc. See advertisement.

*Bound Volumes.*—Subscribers can have their back volumes bound here neatly at $1.00: the postage is about 17 cents on the bound volume. It can be lessened by tearing out the advertisements in mailing to us. The volume for 1879 makes a very pretty book and will make a very useful book of reference containing formulæ and suggestions adapted to almost every form of disease.

The subscriber who neglects to bind his journals does great injustice to himself and his library.

*Southern Medical College.*—This new Institution has proven, in all respects, a remarkable success. The number of matriculates surpassed the expectations of the most sanguine, exceeding that of any former medical institution in the South. The curriculum of study has covered a wide field, and the course of lectures yet going on are highly satisfactory to the students in attendance.

The many medical gentlemen who have visited the Institution during the session have spoken in very complimentary terms of the ability and thoroughness with which the various professorships have been filled, and predict for it a brilliant future.

In our next, or March number, we will give an account of the first commencement exercises, etc., of the Institution.

*Sixth Decennial Pharmacopeia Convention.*—By virtue of authority devolved upon me as the last surviving officer of the Pharmacopeia Convention of 1870, I again call the attention of "the several incorporated State medical societies, the incorporated medical colleges, the incorporated colleges of physicians and surgeons and the incorporated colleges of pharmacy throughout the United States," to the importance of appointing delegates to the Sixth Decennial Pharmacopeia Convention, and of sending the names and residences of the same to me for publication. The Convention meets on the first Wednesday in May, 1880, and I am required "to publish the names and residences of the delegates, for the information of the medical public, previous to its meeting."

I have received so far the names and residences of the following delegates :

From the Massachusetts College of Pharmacy, Boston : Prof. G. F. H. Markoe, Ph.G.; Samuel A. D. Sheppard, Ph G.; Thomas Doliber, Ph.G.

From the Philadelphia College of Pharmacy : Prof. John M. Maisch, Alfred B. Taylor, Prof. Jos. P. Remington.

From the Louisville College of Pharmacy : Prof. Emil Scheffer, Prof. C. Lewis Diehl, E. Vincent Davis.

From the Maryland College of Pharmacy, Baltimore, Md.: *Delegates*—Wm. S. Thompson, Louis Dohme, Jos. Roberts. *Alternates*—Charles Caspari, Jr., Dr. John F. Moore, Dr. Robert Lautenbach.

From the Medical Society of the District of Columbia: Prof. D. W. Prentiss, M.D., Prof. Thomas Antisell, M.D., Emeritus Prof. James E. Morgan, M.D.

From the National Medical College of Columbia University, Washington, D. C.: Prof. W. W. Johnston, M.D.; Prof. D. W. Prentiss, M.D.

From the Medical Department of the University of Georgetown, D. C.: Prof. W. H. Ross, M.D.; Prof. C. H. A. Kleinschmidt, M.D., and Dr. S. C. Busey.

From the National College of Pharmacy, Washington, D. C.: Mr. W. S. Thompson, Prof. Oscar Oldberg, Mr. R. B. Ferguson.

[Signed] JAMES E. MORGAN, M.D.,
905 E Street, N. W., Washington, D. C.

Washington, D. C., January 28, 1880.

### *METRIC OR DECIMAL SYSTEM.*

The following simple table gives all that there is in the metric or decimal system of weights and measures :

MONEY.

10 mills make a cent.

10 cents make a dime.

10 dimes make a dollar.

10 dollars make an eagle.

LENGTH.

10 milli-meters make a centimeter.
10 centi-meters make a decimeter.
10 decimeters make a meter.
10 *meters make a decameter.
10 deca-meters make a hectometer.
10 hecto-meters make a kilometer.
10 kilo-meters make a myriameter.

WEIGHT.

10 milli-grammes make a centigr.
10 centi-grammes make decigram.
10 deci-grammes make a gramme.
10 †grammes make a decagramme.
10 deca-grammes make a hectogr.
10 hecto-grammes make a kilogr.
10 kilo-grammes make a myriagr.

CAPACITY.

10 milli-meters make a centiliter.
10 centi-liters make a deciliter.
10 deci-liters make a liter.
10 ‡ liters make a decaliter.
10 deca-liters make a hectoliter.

The square and cubic measures are nothing more than the squares and cubes of the measures of length. (Thus, a square and a cubic millimeter are the square and the cube of which one side is a millimeter in length.) The are and stere are other names for the square decameter and the cubic meter.

* A meter is equal to 39.368 American inches.
† A gramme is equal to 16.433 grains troy or avoirdupois.
‡ A liter is equal to 2.113 American pints.—*Ex.*

### *CLIMATE OF GEORGIA.*

One of our subscribers from a Northern state writes to know if the climate of Atlanta is adapted to consumptives. We hesitate not to express the opinion that it is, and that middle Georgia and the entire section of Georgia, particularly the Northeastern portion of the State, is better adapted to consumptives than Florida and some other resorts in a more Southern section—we mean as a permanent place of residence. In the interior of Florida the climate is mild and the hygrometric conditions favorable in the winter. In the summer, however, it is too warm and relaxing, and malaria is too prevalent in most sections of the State to make it safe for the consumptive during the summer months. In Jacksonville and at all points on the coasts of Florida there is too much moisture in the atmosphere to make it desirable at any season. The sea breezes in winter are also exceedingly penetrating and disagreeable. In the interior of the State, among the pines, these objections obtain in much less degree. So in certain localities in Southern Georgia, the climatic conditions are favorable the year round. This is true, especially of Thomasville and Cuthbert, Georgia. These are healthy points the year round, and the climate mild, uniform and free, in a great degree, from malaria, though the long summers become somewhat enervating to some constitutions.

In middle and upper Georgia the summers are not excessively warm, nor are the winters severe, the thermometer seldom falling below 30° in winter or rising above 85° in summer.

We mention as among the desirable points in upper Georgia for permanent residence of consumptives the cities of Griffin, Atlanta, Marietta, Gainesville, Athens, Madison and the villages and country adjacent to these points.

---

### *DISSECTING MATERIAL.*

A great deal of overwrought and injudicious sensation has been kept up by the newspapers in Atlanta, relative to the procurement of dissecting material; it being charged upon the colleges that they were grave robbing in this vicinity. The janitor of the Atlanta Medical College was arrested, threatened by the mob and finally convicted of robbing a grave in a neighboring county, although upon search the authorities failed to find the remains in their possession.

We can say for the *Southern Medical College*—the institution with which our editorial corps is connected, that the Faculty do not desire or intend to procure subjects in this vicinity, or in any way that may be objectionable even to the most sensitive and fastidious in such matters; on the contrary they instructed their Demonstrator, early in the session, not to do so, and to procure only material from morgues and pauper grounds in distant sections where they can be obtained without let or hindrance. They are pleased to say to the Profession and to Students everywhere that dissecting material can and will be abundantly secured, and that the facilities for the study of practical anatomy in the Southern Medical College will be equal to the best in the country.

---

### *LITTLE THINGS IN PRACTICE.*

There are many practical and useful ideas which from their very simplicity are overlooked in emergencies. One of these we now mention as suggested by a recent occurrence in practice.

A very fleshy and clumsy lady being thrown from a wagon received a severe injury to her back, so that the least motion caused excruciating pain. The accident occurred in the public road, half a mile from her residence. How to carry her home with the least injury and suffering was the first thought. A buggy was brought, but the effort to lift her into it seemed almost impracticable and the pain too excruciating to be borne, and a like trouble must be encountered in getting her out. At length a large armed rocking chair was brought, into which she was placed with comparatively little pain, her back finding support on a pillow, a half-reclining position being secured by throwing back the chair and supporting her feet. A hand spike was then placed under the chair long enough for two men to take hold on each side, while one man supported the back of the chair, and she was thus carried to her home with comparative ease and convenience.

### *RECEIPTED.*

[Receipts not acknowledged privately are entered here.]

1879.—J. H. Payne; R. B. Stapleton; J. W. Talley; J. E. Pope; Jno A. Gordon; R. W. Lovett; P. M. Catching; J. S. L. Miller; I. H. McMullan; J. M. Boring.

1880.—M. L. Barron; J. W. Baker; W. E. Bryant; A. A. Davidson; W. H. Bledsoe; J. R. Harrison; T. L. Doster; G. M. Patterson; R. E. Hutchins; H S. Shiell; C. H. Jones; T. S. Parham; D. B. Hamilton; C. S. Priestly; C. P. Sanders; A. T. Park; W. P. Wright; Jas. S. Glenn; D. S. Aswell; Allison Lockwood; L. J. Bachman; Z. J. Scott; W. J. McAlpin; C. J. Burrough; R H. Edwards; N. G. West; D. B. Searcy; E. A. Cox; J. C. Webb; J. W. Ethridge; J. M. Pierce; J. C. Wallis; J. B. Foster; L. B. Weathers; J. H. M. Sykes; W. Culpepper; E. W. Austin; Joseph Underwood; O. W. Owensby; John Riches; J. W. Thomas; L. R. Branch; I. Dillworth; Hays & Bro.; L. S. Brownlee; R. A. Cloptor; W. T. Foute.

